# The meaning of spikes from the neuron’s point of view: predictive homeostasis generates the appearance of randomness

**DOI:** 10.3389/fncom.2014.00049

**Published:** 2014-04-29

**Authors:** Christopher D. Fiorillo, Jaekyung K. Kim, Su Z. Hong

**Affiliations:** Department of Bio and Brain Engineering, Korean Advanced Institute of Science and Engineering (KAIST)Daejeon, South Korea

**Keywords:** Bayesian, Jaynes, inference, prediction, neural code, noise, stochastic, random

## Abstract

The conventional interpretation of spikes is from the perspective of an external observer with knowledge of a neuron’s inputs and outputs who is ignorant of the contents of the “black box” that is the neuron. Here we consider a neuron to be an observer and we interpret spikes from the neuron’s perspective. We propose both a descriptive hypothesis based on physics and logic, and a prescriptive hypothesis based on biological optimality. Our descriptive hypothesis is that a neuron’s membrane excitability is “known” and the amplitude of a future excitatory postsynaptic conductance (EPSG) is “unknown”. Therefore excitability is an expectation of EPSG amplitude and a spike is generated only when EPSG amplitude exceeds its expectation (“prediction error”). Our prescriptive hypothesis is that a diversity of synaptic inputs and voltage-regulated ion channels implement “predictive homeostasis”, working to insure that the expectation is accurate. The homeostatic ideal and optimal expectation would be achieved when an EPSP reaches precisely to spike threshold, so that spike output is exquisitely sensitive to small variations in EPSG input. To an external observer who knows neither EPSG amplitude nor membrane excitability, spikes would appear random if the neuron is making accurate predictions. We review experimental evidence that spike probabilities are indeed maintained near an average of 0.5 under natural conditions, and we suggest that the same principles may also explain why synaptic vesicle release appears to be “stochastic”. Whereas the present hypothesis accords with principles of efficient coding dating back to Barlow (1961), it contradicts decades of assertions that neural activity is substantially “random” or “noisy”. The apparent randomness is by design, and like many other examples of apparent randomness, it corresponds to the ignorance of external macroscopic observers about the detailed inner workings of a microscopic system.

## Introduction

Within the field of information theory, there is a common though often implicit belief that the information content of a signal depends only on its statistical relationship to the quantity of interest (e.g., Rieke et al., 1997). In this view, we should be able to understand the meaning of a neuron’s spikes entirely through an input-output (I-O) analysis, without any consideration of events happening within the neuron. This view of information is a natural consequence of the opinion that probabilities are essentially equivalent to frequencies and are properties of an observed system, rather than being entirely conditional on the knowledge possessed by an observer about that system (Fiorillo, 2012). The faults and limitations of the “frequentist” definition of probability and information within neuroscience have been discussed previously, together with the virtues of the “Bayesian” definition advocated by Jaynes (Fiorillo, 2012). The present work considers a neuron as an observer that knows only what is in its internal biophysical state. Like Jaynes (2003), we presume that probabilities are always conditional on the local and subjective information of an observer, through the universal and objective principle of logic (Fiorillo, 2012).

At the center of the present work is a very simple idea that has been around at least since the advent of information theory (Shannon, 1948). If an event has two possible outcomes, observation of the outcome will convey the maximum amount of information if the probability of each is equal prior to observation. The output of most neurons is a spike that is virtually all-or-none (unlike a graded output, this allows for reliable communication across distances), and thus the amount of information that could be conveyed by a spike will be maximal when its probability is 1/2. Although this is not controversial, it has nonetheless been associated with considerable confusion. This is because the information conveyed by any event will necessarily depend entirely on the prior knowledge of the observer, and a signal that is highly informative to one observer could be entirely uninformative to another. Without a clear understanding of how probabilities and information depend on the observer, scientists have often misattributed their information to the neurons they study (Fiorillo, 2012).

Consider the case of selecting questions that must have “yes” or “no” answers (as in the game “20 Questions”). If there is a real number known to be within the range of 0–10, and the goal is to guess it as accurately as possible after receiving the answer to a yes-no question, then the best strategy would be to bisect the range of possible numbers evenly by asking “is the number greater than 5?” This is the optimal question because “5” is the expected value and will maximize the questioner’s uncertainty (entropy) by causing both “yes” and “no” to have equal probabilities of 1/2. The answer will therefore provide the maximal amount of information by eliminating this uncertainty. Any other question would result in less initial uncertainty about the answer, and therefore the answer would convey less information. However, the answer is informative only if one knows the question. A second observer may also know that the answer will be “yes” or “no”, but not know the question. Thus two observers could agree with one another that the probability of “yes” is 1/2, but the one who knows the question could get maximal information, whereas the one who is entirely ignorant of the question would learn nothing useful from the same answer, and might be tempted to declare that the answer was entirely random noise. Here we suggest that in many instances neuroscientists have essentially taken the perspective of the naive observer, being ignorant of the prior information of the neuron that “asks the question” and generates and interprets the spikes.

It is well known that the spike output of neurons often appears “stochastic”, insofar as the same stimulus input causes a variable spike output. Likewise, when an action potential arrives at a synaptic terminal, it may or may not causes release of neurotransmitter. Although it has been noted that this variability in I-O relations could convey information (Softky and Koch, 1993; Softky, 1995), over decades it has repeatedly been attributed to “noise” or “randomness” that is presumed to degrade the ability of neurons to transmit information (Calvin and Stevens, 1968; Tolhurst et al., 1983; Shadlen and Newsome, 1994, 1998; London et al., 2010). Models of how neurons could perform inference have proposed that the variability may serve a function by signifying subjective uncertainty (Pouget et al., 2000; Deneve et al., 2001; Hoyer and Hyvarinen, 2003; Jazayeri and Movshon, 2006; Ma et al., 2006; Beck et al., 2008; Berkes et al., 2011). Whether the variability is noise or a functional signal related to uncertainty, uncertainty (ignorance) itself is not desirable, and both of these viewpoints propose that a more variable output (in response to a fixed input) would correspond to greater uncertainty. In contrast, a central conclusion of the present work is that this same variability is a signature of optimality and knowledge if we consider the perspective of a neuron, which performs work (consumes energy) to produce it.

It may seem strange or even unscientific to treat neurons as observers. Indeed, Skinner and other “behaviorists” argued that to be objective and to give psychology a firm scientific basis, we should not even treat humans and other animals as observers, but that we should instead characterize their behavior solely in terms of I-O relations (environmental inputs and behavioral outputs). Psychology has now mostly rejected that view, and instead seeks to understand mental events and behavior from the first-person perspective of the brain as it observes its world. The probability theory of Jaynes (2003) allows us to describe a subjective state of knowledge in an objective manner (Fiorillo, 2012). Many elegant studies have now demonstrated that aspects of perception, cognition, and motor control that appeared maladaptive can be understand as optimal (rational or Bayesian) if we take the brain’s perspective, including sensory illusions (e.g., Weiss et al., 2002; Niemeier et al., 2003; Yang and Purves, 2003; Körding and Wolpert, 2004).

Illusions would seem to provide clear evidence of the brain’s malfunction. However, the brain only appears to function poorly from the perspective of a third-party observer who knows something that the brain itself does not and could not know. Whereas the brain must infer the sensory stimulus given limited sensory evidence and its own prior knowledge of what is likely, the third party has privileged knowledge of the “true sensory stimulus”. The brain integrates its information as well as it possibly can, but it nonetheless “misperceives” the stimulus on those rare occasions when the stimulus is something unusual and unexpected, like a fast moving object (Weiss et al., 2002). Thus, what once appeared pathological from our perspective as external observers can suddenly be understood as optimal once we consider the unique perspective of the neural observer. We propose that, in a precisely analogous manner, the variability in a neuron’s I-O relationship is optimal rather than pathological once one takes the neuron’s perspective.

## Information theory from the neuron’s perspective

**Figure 1:**
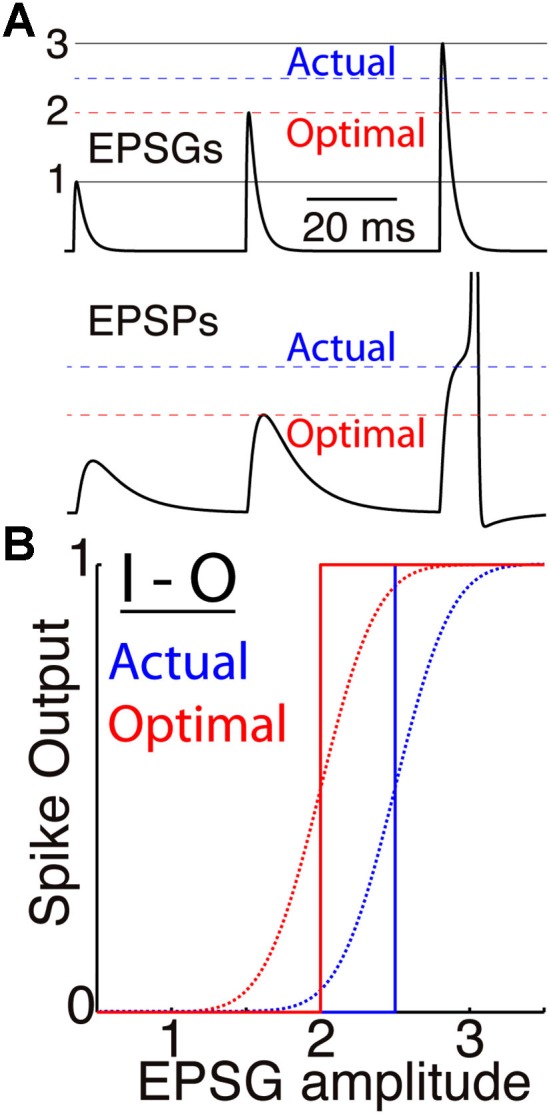
**Membrane excitability and the I-O relation**. **(A)** A simulated neuron receives EPSGs (top) of only three amplitudes (1, 2, and 3 units) with intervals long enough so that there is no temporal summation of EPSPs (bottom). It generates a spike only if EPSG amplitude is 2.5 units or greater (dashed blue line), and Hypothesis 1 states that 2.5 is therefore the expectation from the neuron’s perspective. If its excitability were optimal (given knowledge of the 3 EPSG amplitudes but no means to know which would occur at a particular moment), its spike threshold would correspond to 2.0 units (dashed red line). **(B)** Spike output as a function of EPSG input amplitude, for the neuron illustrated in “**A**” (blue) and for the optimal neuron (red). Step functions (solid lines) correspond to binary spike output, and sigmoid functions (dotted curves, with arbitrarily chosen slopes) to spike probability (SP) as measured from the perspective of an external observer who does not know excitability or EPSG amplitude.

Our theory is closely related to Barlow’s theory of “efficient coding” (Barlow, 1961) and the extensive literature that followed on the relation between neural activity and natural stimulus statistics (e.g., Laughlin, 1981; Srinivasan et al., 1982; Linsker, 1988; Rieke et al., 1997; Stemmler and Koch, 1999; Brenner et al., 2000; Simoncelli and Olshausen, 2001; Hosoya et al., 2005; Sharpee et al., 2006). In particular, the frequency distribution of a neuron’s output (e.g., firing rate) should be “matched” to the distribution of its input intensity, so that if the input distribution is Gaussian, then the I-O relation should ideally follow the form of the corresponding cumulative Gaussian (Laughlin, 1981; Brenner et al., 2000). A neuron’s membrane excitability determines its I-O relationship, and excitability should adapt so that the location (*x*-offset) and scale (slope) of the I-O curve cause the neuron’s output to be most sensitive to the most probable inputs (the steep and linear portion of the curve) at the expense of improbable inputs (the non-linear and nearly flat portion of the curve). Figure 1 illustrates this principle as it applies to the cellular level, with the amplitude of excitatory postsynaptic conductance (EPSG) as input and a binary spike or no spike as output. We refer to the principle as “predictive homeostasis”, rather than “predictive” or “efficient” coding, to emphasize that it is implemented by numerous homeostatic mechanisms of the same sort that are well known by biologists to be present in all cells.

Efficient coding by a neuron should ideally have the effect of maximizing the Shannon information in its spike output (*I_S_*) about its excitatory synaptic input (*E_EPSG_*). This information would be equal to the reduction in entropy (*H*) caused by observation of the spike output (*S*).

(1) $$I_{S}(E_{EPSG}|E_{X}S)=H(E_{EPSG}|E_{X})-H(E_{EPSG}|E_{X}S)$$

The “prior entropy” [*H*(*E_EPSG_*|E_X_)] would be the width or uncertainty of the probability distribution, which would be reduced by observing spike output (S). We equate a neuron’s membrane excitability with the prior information (*E_X_*). Unfortunately we are unable at this time to derive the prior probability distribution [*p*(*E_EPSG_*|E_X_)], as discussed previously (Fiorillo, 2012). However, since spike output (*S*) is binary, its information content is maximized when its prior probability [*p*(*S|E_X_*)] is 1/2. This corresponds to that case that it indicates whether the input is more or less than the prior expectation (*<E_EPSG_*>|E_X_), so that observation of spike output reduces the prior entropy [*H*(*E_EPSG_*|E_X_)] by 1/2 (assuming the prior distribution is symmetric). We propose below that this is precisely the meaning of a spike from the neuron’s point of view.

There are at least three respects in which the present hypothesis goes beyond previous conceptions of this principle. First and of greatest importance, we consider the neuron’s point of view, by which we mean that the expectation of interest is entirely conditional on the neuron’s prior information as inherent in its membrane excitability (*E_X_*). This contrasts with the conventional approach in which probabilities are conditional on the knowledge of an external observer (e.g., Rieke et al., 1997). Although the importance of “taking the neuron’s point of view” has been recognized previously (Rieke et al., 1997), it has virtually never been done for reasons described elsewhere (Fiorillo, 2012). It requires that we set aside whatever knowledge we might have about the neuron’s input (such as knowledge of the frequency distribution of its inputs, commonly referred to as “the stimulus ensemble”) and its statistical relationship to the neuron’s output. Instead, we consider only the information that we believe to be contained within the physical structure of the neuron or other observer.

A second distinction from most previous work is in considering the millisecond timescale, in which spike output is necessarily binary. Most previous work on efficient coding, including our own (Fiorillo, 2008), considered output to be analog firing rate or membrane voltage (Laughlin, 1981; Linsker, 1988; Stemmler and Koch, 1999; Brenner et al., 2000; but see Deneve, 2008a). However, all the relevant membrane properties evolve on the scale of 1 ms or even less (Softky, 1995), including synaptic conductance and the action potential itself. There are many slower processes as well, some of which effectively average over spikes, and these are also incorporated within the theory (Fiorillo, 2008). But to adequately capture the informational dynamics, and especially the proximal cause of spikes, we must consider the fastest time scale in which membrane properties vary. Our approach rests on the intuitive notion that information is inseparable from matter, energy, and causation, so that if the charge distribution across a neuron’s membrane is changing within a millisecond, so is the information that it contains.

Third, like Stemmler and Koch (1999), we focus on a single neuron at the cellular level, so that input and output are both within the neuron. This contrasts with most previous work on efficient coding in which input corresponds to external sensory stimuli, and thus multiple neurons intervene between input and spike output. The cellular approach has several advantages. First, as a simpler system, analysis of a single neuron better allows us to identify mechanisms by which neurons predict their input and adapt their I-O relation (e.g., Hong et al., 2013). Second, whereas efficient coding is often thought of as a “sensory problem”, the cellular analysis emphasizes the general nature of the problem, since all spiking neurons are faced with the problem of using a binary output to efficiently represent an analog input. Third, the inference made by a neuron or any observer must be based entirely on local information, as must all learning. Compared to a network of neurons, it is relatively easy to imagine how learning or natural selection might optimize I-O relations of single neurons (Fiorillo, 2008). If a single neuron can optimize its local I-O relation, a feedforward series of such neurons, each adapted to the local statistics of its input, can be expected to approximately optimize the systems-level I-O relation (between external sensory stimuli and spike output).

### A previous model of prediction by a single neuron

A previous theory had the goal of providing a general informational account of the nervous system (Fiorillo, 2008). Although narrower in scope, the present work builds on the previous framework. In both cases, a neuron’s membrane compares the intensity of its sensory related input to its expectation, and its output signals “prediction error”. As in other models of neuronal prediction error (e.g., Schultz et al., 1997; Izhikevich, 2006; Frémaux et al., 2010), the homeostatic null point was presumed to correspond to “baseline firing rate” or an intermediate membrane voltage “just below” spike threshold (Fiorillo, 2008). The null point and its neural basis was not precisely defined or justified. Indeed, the notion of a “firing rate” is itself problematic, since it is not a physical entity that exists at any particular point in space and time. The present work provides a solution by considering the timescale of a single spike and proposing that the threshold voltage for a spike corresponds to the homeostatic point at which the prediction error is 0.

Information necessarily has both a qualitative and quantitative component, and the earlier theory dealt with both (Fiorillo, 2008). Like Shannon (1948), we deal here only with the quantitative aspect. Since EPSGs are the proximal cause of spikes, the set of excitatory synapses will determine the qualitative nature of the information within spikes by specifying the neuron’s receptive field, or “stimulus” (for example, light of certain wavelengths in some region of space). Previous work suggested how learning could shape a receptive field to insure that it contributes information of biological importance (e.g., Schultz et al., 1997; Izhikevich, 2006; Fiorillo, 2008; Frémaux et al., 2010). Here we address only the issue of how membrane excitability can enable spike output to best preserve (maximize) information about EPSG input (the “coding strategy”), regardless of the meaning and importance of that input (and “what it should code”).

## Theory of predictive homeostasis

After specifying a simple biophysical model of membrane excitability, we proceed to describe its information content based only on its physical properties (Hypothesis 1), and then to prescribe excitability based on biological optimality (Hypothesis 2).

### The biophysical model

We assume a generic neuron that follows established biophysics. It has a spike generating mechanism as well as two conductances, an EPSG and an “intrinsic” conductance. In general, each of these will vary over time, and will be the sum of multiple individual conductances (synapses or types of ion channel). EPSGs are discrete events consisting of one or more unitary EPSGs, where a unitary EPSG results from a spike in a single presynaptic neuron (which releases at least one vesicle of neurotransmitter, and usually more). Unitary EPSGs vary in amplitude over time, even in the case that they derive from the same presynaptic neuron (due to variable numbers of vesicles being released across multiple release sites). In addition, unitary EPSGs and their associated EPSPs will display spatial and temporal summation. Although, they are discrete events and may occur only sparsely over time, they convey information about continuously varying external variables, such as light or sound intensity. We focus on the amplitude of EPSGs because although they are internal to a neuron, their timing and amplitude depend almost entirely on variables external to the cell.

**Figure 2:**
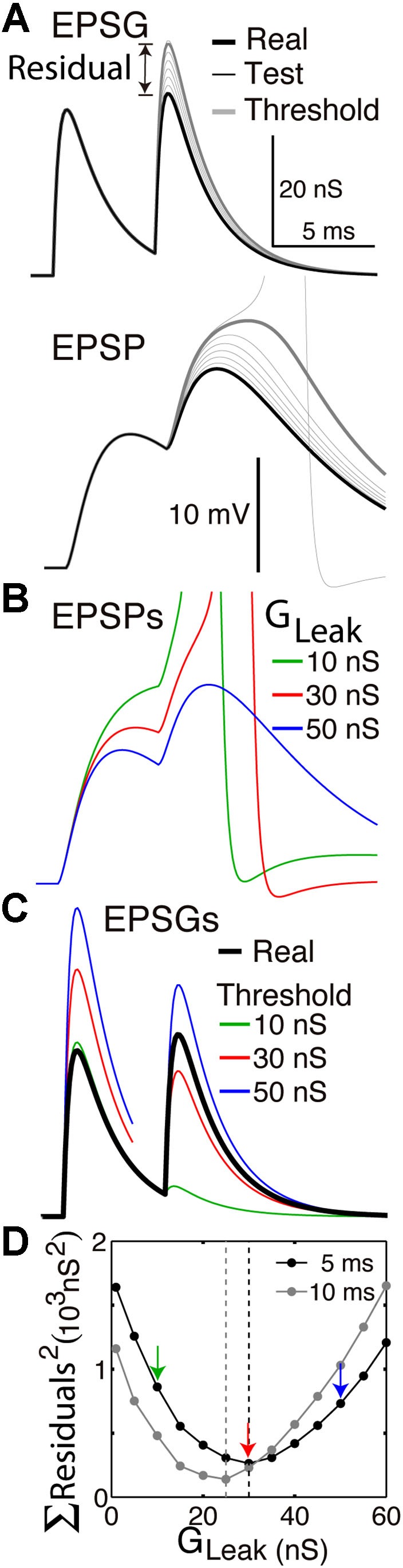
**Finding the optimal homeostatic conductance**. A neuron with only a leak conductance (reversal at −70 mV) and spike mechanism was simulated using NEURON software. **(A)** The method of measuring distance from optimality. Top, the neuron received two EPSGs of equal amplitude (30 nS) separated by a 5 ms interval (thick black). At onset of each real EPSG, test ESPGs (thin black, shown only for the second) of varying amplitudes were applied to find the “threshold EPSG” (thick gray) for which the EPSP peak (bottom) is precisely at spike threshold. The “residual” is the difference in peak amplitude of the real and threshold EPSG, and it measures the distance of excitability from optimality. **(B)** EPSPs generated by the real EPSGs in “**A**”, but with leak conductances of 10, 30, and 50 nS. **(C)** Threshold EPSGs for the same three leak conductances. The 10 nS conductance best minimized the residual for the first EPSG, but the sum of the two squared residuals is less for the 30 nS conductance. **(D)** The sum of squared residuals was minimized by leak conductances of 30 and 25 nS in the case of 5 and 10 ms inter-EPSG intervals, respectively.

Whether an EPSG arriving at a synapse causes a spike to be initiated near the soma depends on many well known factors, including initial membrane voltage, axial resistance, and membrane capacitance and conductance. Voltage-regulated ion channels change membrane conductance to amplify or suppress an EPSP, even during its brief rise time (∼1 ms). The summed effect of all of these factors is encapsulated by the term “excitability”. We define it here as the energy barrier (*E_X_*) which separates an EPSG from spike threshold, although to be consistent with common usage we would say that a lower energy barrier (a smaller distance from spike threshold) corresponds to a higher excitability. A spike will occur only if the energy in an EPSG (*E_EPSG_*) exceeds the barrier (*E_X_*). Thus excitability is the amount of work that an EPSG must perform to cause a spike, and this corresponds to the amount of charge that must be transferred from the synapse to the site of spike initiation. This could be calculated in principle given a detailed model, but it can be found more easily by injecting “test EPSGs” of incrementally increasing amplitude until spike threshold is reached (Figure 2A). The amplitude of the “threshold EPSG” that is found in this way has precisely the energy *E_X_* that it is needed to reach spike threshold.

### Describing a neuron’s excitability and expectation

Here we equate “the observer” with “membrane excitability”. The excitability at time *t* will determine whether an EPSG with onset at time *t* will cause a spike. What is the expectation of EPSG amplitude given only excitability? Some would answer that this question cannot be answered unless one first observes EPSG amplitudes and thus has knowledge of the frequency of various amplitudes. However, we follow the probability theory of Jaynes (commonly referred to as “Bayesian”), according to which one “sample” of any quantity can provide an expectation of another (through logic, as expressed in the principle of maximum entropy) (Jaynes, 2003; Fiorillo, 2012). From this we presume that knowledge of one mass can be used to estimate another mass, one energy can be used to estimate another energy, etc. More information is always better for a biological observer, but an observer simply “knows what it knows”.

The EPSG performs work to drive membrane voltage towards spike threshold, and excitability works against the EPSG. In the absence of any information beyond excitability itself, the probability that an EPSG will cause a spike is 1/2 (based on logic, the maximum entropy principle), and thus the expectation (*<E_EPSG_>*) must be equal to the excitability (*E_X_*) (equation 2) (by the mathematical definition of “expectation”).

*Hypothesis 1: A spike is generated only if EPSG amplitude is greater than expected given the information in membrane excitability (equation 2)*.

(2) $$<E_{EPSG,t+r}>\;|E_{X,t}=E_{X,t}$$

Equation 2 refers to the expectation (at time *t*) of the peak amplitude of an EPSG if one were to start at that same time (which would reach its peak at time *t + r*, where *r* refers to the stereotyped rise time of an EPSG, typically about 0.5 ms). Thus, we are referring to an expectation of a potential future event. When excitability is low, the energy barrier (*E_X_*) is high, and a large EPSG is expected. The expectation is just the “center” of a probability distribution, and we are not speculating here about the uncertainty (width) of the distribution. Because spike threshold corresponds to the expectation, a spike conveys maximal information (relative to any other relation between expectation and spike threshold, assuming that the prior probability distribution is symmetrical). A spike would not convey any information beyond that in membrane voltage at the spike initiation site at the time of its generation, but it would reliably communicate that information throughout the entire neuron.

Given an EPSG with onset at time *t*, it is useful to denote membrane excitability at time *t* as “prior information” to distinguish it from the new information in the EPSG. Hypothesis 1 is essentially just that a spike signals “prediction error”. Prediction errors are known to be efficient and useful signals, but there is not much intelligence in a prediction error if the prediction itself is not accurate. Our use of “prediction error” is merely descriptive and could be applicable to a large variety of physical entities.

A traditional balance scale provides a useful analogy. It consists of an arm that rotates around a central joint depending on a known reference weight on the left (excitability) and an unknown weight on the right (the EPSG). The arm rotates continuously (over some range) as a function of the difference between the two weights (membrane voltage). The scale generates a binary output to signal which weight is greater (right side up or down, analogous to a spike). Prior to placement of the unknown weight on the right side, the expectation of the unknown weight would be equal to the known weight, and “right side up” (a spike) would indicate that the unknown weight was greater than the expectation.

Our challenge here is merely to describe the information in a physical entity based on physics and logic. Unlike most of biology, physics is purely descriptive insofar as it is agnostic to “how the world should work”. In describing the knowledge within a balance scale or a neuron, we do not presume that the reference weight or the excitability is well suited for measuring the unknown weight or EPSG. Rather we presume that it is the only information possessed by the observer.

### Critique of hypothesis 1

We believe that an observer is something remarkably simple. Excitability is simple insofar as it is just a single number at a single moment in time. We presume that knowledge of excitability does not include knowledge of its constituents, not even membrane voltage and conductance. Furthermore, excitability is entirely internal to a neuron, and therefore it could be said that we are “taking the neuron’s point of view”. However, we also believe that a single observer should correspond to information that is physically integrated in space and time. Membrane voltage fits this definition, summing currents over a local region of membrane. By contrast, excitability includes multiple factors in addition to membrane voltage. Even though those factors are all present in a small and well defined part of a neuron, they are not being physically summed together. Therefore we infer that excitability corresponds to information distributed across more than one observer, and Hypothesis 1 falls short of our ideal of “describing the knowledge of an observer”. It remains for future work to describe the knowledge in membrane voltage about EPSG input.

Despite its limitations, we conclude that Hypothesis 1 represents a useful default hypothesis by virtue of its simplicity. For the expectation of EPSG amplitude given excitability to be other than excitability itself, and the prior probability of a spike to be other than 0.5, would require additional information that we have not assumed. We assume that our observer has knowledge, but it is an absolute minimal level of knowledge. The prescriptive hypothesis presented below does not depend upon the veracity of this descriptive hypothesis. However, the simple logic of this descriptive hypothesis suggests that a neuron does not need any particular design in order for it to have an expectation, and for its spikes to be maximally informative from its perspective. Hypothesis 1 states that a known is the best guess of an unknown, whereas Hypothesis 2 states that biology works towards the ideal in which the known is in fact equal to the unknown.

### Prescribing a neurons excitability and expectation

Hypothesis 2 expresses our belief that the excitability of a healthy adult neuron will tend to accurately predict EPSG amplitude, because it has been shaped by both natural selection over generations and associative learning rules. An accurate analog expectation of an analog variable will be too high in half of cases, and too low in the other half.

*Hypothesis 2: Expectations will be accurate under natural conditions, and therefore half of EPSGs will cause spikes*.

The perfect match of EPSG amplitude to its expectation is not attainable, and even if it were, the spike output must be 0 or 1, corresponding to a negative or positive prediction error. The optimal expectation would therefore result in a spike in response to precisely half of EPSGs (Figure 1). The spike probability (SP) of 1/2 given the neuron’s prior information (Equation 2) would then match the frequency distribution of spikes given EPSGs. Although this optimal neuron would have low subjective uncertainty (which we do not attempt to quantify here), its spikes would appear to be random to an external observer who is ignorant of variations in excitability and EPSG amplitude.

Hypothesis 1 proposes that spikes are maximally informative simply as a consequence of logic and physics, regardless of the prior information in excitability. Hypothesis 2 implies that accurate prediction (or minimizing total entropy) is an important biological goal, and it will be aided by accurate prior information (a low prior entropy, [*H*(*E_EPSG_*|E_X_)]. As a contrived example, neuron “A” has prior knowledge that EPSG amplitude must be between 0 and 10, and neuron “B” knows that it must be between 4 and 6. If the expectation of each neuron is 5, then a spike would tell neuron “A” that the actual amplitude must be between 5 and 10, whereas it would indicate to neuron “B” that it is between 5 and 6. Neuron “B” would have more total information given its prior information and a spike [less “posterior” entropy, *H*(*E_EPSG_*|E_X_S)].

## Measuring the accuracy of a neuron’s predictions

Here we propose methods by which we can measure the accuracy of a neuron’s expectation. The neuron itself also needs to assess the accuracy of its predictions, but we address this in a later section (Prediction Errors in Learning). We use “accuracy” to refer to a distance between two expectations, the more certain of which could be called “reality” or the “actual” or “true” value, and the less certain “the expectation”. Thus “accuracy” is not the same as uncertainty (entropy). We have not attempted to quantify uncertainty, but greater uncertainty would naturally tend to be associated with less accuracy.

### Residuals

The best method for us to assess the accuracy of expectations will depend on our ability to know and control the neuron. If we have complete knowledge and control, as in a Hodgkin-Huxley model, then it is easy to measure excitability by injecting “test EPSGs” of varying amplitude to find the “threshold EPSG” amplitude that causes an EPSP to reach precisely to spike threshold (Figure 2A). This “threshold EPSG” is our measure of excitability (*E_X_*) and corresponds to the neuron’s expectation of EPSG amplitude (Hypothesis 1). The “residual” EPSG amplitude (*E_res_*) measures the difference between the neuron’s expectation (*E_X_*) and the actual EPSG (*E_EPSG_*) (Figure 2A).

(3) $$E_{res}=E_{EPSG}-E_{X}$$

Indices for time have been omitted for simplicity. In principle, excitability could be measured at each instant (“real time”). However, it is not an expectation of EPSG likelihood, but of EPSG amplitude if one were to occur. Thus the expectation is only put to the test when an EPSG occurs. A high level of inhibitory conductance would correspond to expectation of a large EPSG, but if none occurs, the cost would be only metabolic and not informational. Thus we measure excitability only at the time of real EPSGs (onset of test and real EPSGs are always synchronous) (Figure 2A).

A residual (*E_res_*) will be positive if the EPSG causes a spike, and will indicate the extent to which the EPSG was greater than expected and excitability was too high (*E_X_* was too low). It will be negative if EPSG amplitude was less then expected, indicating that excitability was too low (*E_X_* was too high). If a neuron is making accurate predictions, the residuals will be small with an average of 0, and spike generation will be highly sensitive to small variations in EPSG amplitude, as experiments have demonstrated in some cases (Carandini, 2004; Kuenzel et al., 2011).

The residual is an error of sorts, and an objective of learning would be to modify homeostatic conductances so that residuals are minimized. However, we are not suggesting that a neuron has any direct means of knowing the residuals, and we reserve the term “prediction error” to describe errors from the neuron’s point of view (as signaled by spikes). Thus the residuals cannot drive learning, and we present them only as a means for us to assess the accuracy of a neuron’s predictions (its distance from optimality) (Figure 2).

### Spike probability from the experimenter’s point of view

**Figure 3:**
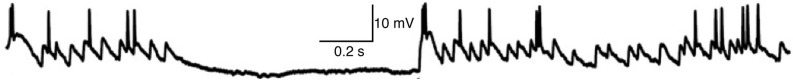
**Whole-cell recording of membrane voltage from a thalamocortical neuron in LGN of anesthetized cat during visual stimulation with a naturalistic movie (adapted with permission from Figure 2 of Wang et al., 2007)**. The hyperpolarized period without EPSPs corresponds to opponent inhibition (caused by light in the receptive field of this OFF-type neuron), whereas homeostatic “same-sign” synaptic inhibition tends to occur at nearly the same time as synaptic excitation and thus is not visible here (Wang et al., 2011).

Hypothesis 2 states that predictions should be accurate under natural conditions in a normally functioning brain. Even with *in vivo* intracellular recording (Figure 3) it is challenging to estimate EPSG amplitudes and excitability. It is therefore useful to assess the accuracy of a neuron’s expectations given only knowledge of the time of EPSG inputs (or presynaptic spikes) and spike outputs. For this purpose we define *spike probability* (SP):

*Spike probability (SP): The probability that at least one output spike is caused by an EPSG at a “driving” synapse*.

SP is defined to incorporate knowledge that at least one input spike and associated EPSG has occurred at a “driving” synapse (see Driving EPSGs as the Cause of Spikes), and that if the EPSG causes a spike, it will occur within a certain delay (generally less than 5 ms in a typical neuron). A unitary EPSG could be the sole cause of a spike or a partial cause together with other EPSGs that are nearly synchronous. SP does not include knowledge of excitability or the specific amplitude of the EPSG (since that information is seldom available from experimental data). Thus, our uncertainty about whether or not a spike will occur is not because there is anything intrinsically “random” about the occurrence of spikes, but because we are ignorant of the “black box” that is EPSG amplitude and membrane excitability.

SP of 1/2 is the homeostatic ideal and will cause spike output to be maximally sensitive to small gradations in EPSG amplitude (Figure 1). However, we presume that it is a virtually impossible goal to achieve, for the same reason that predictions of the future market value of stocks are almost never precisely accurate. We expect that SP varies dynamically from near 0 to 1 under natural conditions, but with an average over time that is near 1/2.

## Implementation of predictive homeostasis

### Driving EPSGs as the cause of spikes

We presume that information follows causation, and that the EPSG is (and should be) the proximal cause of spikes. The neuron’s knowledge is substantially limited to its proximal causes, for the same reason that our conscious perception corresponds to proximal sensory causes in the immediate past, which we can infer with relatively little uncertainty, but not the innumerable distal causes further in the past. In a typical neuron the proximal cause of a spike would be an EPSG with onset about 0.5–5 ms earlier, mediated primarily by AMPA-type glutamate receptors (Figure 3). We propose that slow membrane depolarization would not be the proximal cause of spikes under natural conditions, including that initiated by offset of synaptic inhibition, activation of G-protein-coupled receptors, and perhaps even AMPA-type glutamate receptors located on distal dendrites (Branco and Häusser, 2011). Excitatory synapses have been classified as either “drivers” or “modulators”, which are distinguished from one another by dendritic location and a variety of other features (Sherman and Guillery, 1998). “Drive” means “cause” in this case, and the drivers alone cause spikes and determine a neuron’s receptive field. In the lateral geniculate nucleus of the thalamus (LGN), “feedforward” retinogeniculate AMPA EPSGs on proximal dendrites are the drivers of spikes, whereas “feedback” corticogeniculate AMPA EPSGs on distal dendrites are “modulatory” (Sherman and Guillery, 1998). The latter could provide “value information” that modulates the strength of driving synapses and thereby changes the shape of a neuron’s functional receptive field (Fiorillo, 2008), but we do not consider its particular function here. “The EPSG” to which we refer is exclusively from “driving synapses”. Slow excitation is naturally more predictable, and predictive homeostasis should work to insure that only fast and large EPSPs cause spikes.

### Mechanisms and timescales

We propose that there are a vast array of homeostatic ion channels and synapses functioning simultaneously on numerous timescales within a single neuron. Each would make a prediction based on a particular recurring pattern of synaptic excitation or inhibition, like those in Figure 3. Homeostatic synaptic inhibition would be dedicated to spatial patterns of activity across neurons (as in the case of “surround inhibition”), whereas voltage-regulated ion channels would be dedicated to temporal patterns (Fiorillo, 2008). To illustrate and test this idea, we need to relate specific homeostatic mechanisms to specific patterns of synaptic drive.

Figure 1 illustrates the case of a neuron that receives EPSGs of only three amplitudes (1, 2, and 3) that are sufficiently separated in time so that there is no temporal summation. Knowing those three amplitudes but nothing else, the optimal expectation is “2” and an EPSG of amplitude “2” should cause an EPSP that reaches precisely to spike threshold (Figure 1). Figure 2 illustrates the case of a neuron that receives excitatory drive from a single presynaptic neuron, with each presynaptic spike causing a unitary EPSG of identical amplitude. Despite the simplification of identical unitary amplitudes, this example remains interesting since temporal summation will cause EPSG and EPSP amplitude to vary with presynaptic inter-spike interval (ISI). This is in fact similar to some real synapses, as discussed further below.

The neuron of Figure 2 is the simplest possible insofar as its only homeostatic conductance is a “leak” conductance, which is voltage-insensitive and constant by definition. If the leak conductance is optimal, it will accurately predict the long-term average EPSG and EPSP amplitude, which vary due to temporal summation. Thus half of unitary EPSGs will exceed average amplitude and cause a spike, and this will only occur when there is sufficient temporal summation (Figures 2A, B). When the neuron’s excitability is “at rest”, the first EPSG in a pair will be of less than expected amplitude and cause no spike, but it will increase excitability so that a second EPSG 5 ms later will cause a spike. We note that even when EPSGs cease and excitability approaches a stable resting level, excitability still provides an expectation of EPSG amplitude, and this could be called the neuron’s “default prior”. Since the amplitude of EPSGs provides information about the intensity of external stimuli, like light intensity, the neuron would always have an expectation of both EPSGs and external stimuli.

Although accurate prediction of the long-term average is important, it would generally be highly inaccurate over shorter periods. Prediction using only a very slow ion channel, like a leak conductance, is analogous to predicting the market value of a stock using only knowledge of its trajectory over the last 10 or 100 years. Better predictions can be made given additional knowledge of any regular pattern, including short term trends. There are a remarkable diversity of voltage-regulated ion channels with a spectrum of kinetic properties, and each of these is proposed to be specialized for making a prediction based on a particular recurring pattern (Fiorillo, 2008). There are obviously many patterns that involve changes in EPSG rate. An increased rate of EPSGs and spikes would activate K+ channels with moderately slow kinetics (such as Kv 7), which would then mediate “spike frequency adaptation” and restore SP towards 0.5 over periods of tens of milliseconds or more.

The rising phases of EPSPs and action potentials are the fastest changes in excitatory drive. Excitability will provide an expectation of an EPSG’s amplitude prior to its onset, but A-type K+ channels activate during the rising phase of an EPSP and thus contribute to excitability as well (in many types of neurons). In principle, the amplitude of an EPSP can be predicted by the slope of its rising phase. If some subtypes of A-type channels inactivate within a millisecond or two (Migliore et al., 1999), they could conceivably exploit this pattern by preferentially suppressing EPSPs with fast rising phases that would otherwise be excessively large (suprathreshold), while inactivating and thereby causing less suppression of slowing rising EPSPs that would otherwise be too small (subthreshold). The amplitude of an individual EPSP could also be predicted given knowledge of the amplitude of a related EPSP (sharing the same cause) in another neuron. This prediction would be mediated by homeostatic synaptic inhibition via GABA_A_ chloride channels, which typically has an onset about 1 ms after EPSG onset (e.g., Blitz and Regehr, 2005), and derives from a neuron with a similar and overlapping set of excitatory drivers (a similar receptive field) (e.g., Wang et al., 2011). GABA_A_ and A-type channels both provide fast homeostatic mechanisms that may “update expectations” within the brief period between EPSG onset and EPSP peak. In addition, it is likely that during a large EPSP (with or without a spike), these channels open and thereby decrease excitability in anticipation of an additional EPSG, since otherwise the sum of the two would greatly exceed spike threshold (Figures 2A–C). It should be noted in this respect that homeostatic mechanisms should be finely tuned not only to excitatory drive but to other homeostatic mechanisms as well. For example, the inactivation of A-type K+ channels could conceivably implement a predictive compensation for GABA_A_ inhibition, since simultaneous activation of both conductances could cause an excessive decrease in excitability.

### Finding the optimal homeostatic conductance

Figure 2 illustrates how we can find the optimal leak conductance for a neuron that has no other homeostatic conductances and receives two EPSGs of equal amplitude that are separated by a 5 ms interval. Because of temporal summation (Figures 2A, B), it is not possible with only a leak conductance to have two EPSPs with both peaks at spike threshold. The best that can be done is for the first to be too small and the second too large (Figure 2C). We presume that the optimal leak conductance is the one that minimizes the sum of the two squared residuals (Figure 2D). If the EPSG interval is 10 rather than 5 ms, there is less temporal summation and thus the optimal leak conductance is smaller (Figure 2D). The optimal conductance in a real neuron would depend on the entire ensemble of EPSG amplitudes that the neuron experiences, and a conductance that is optimal for one pattern will often be suboptimal for another. It is apparent from Figure 2 that if inhibition were increased by an appropriate amount following the first EPSG it would help to restore excitability and thereby make the neuron better prepared for addition of the second EPSG. A neuron with a suitable dynamic homeostatic conductance (like GABA_A_ or A-type channels) would therefore be closer to optimal than any neuron with only a leak conductance.

### Two classes of ion channel

Our earlier work distinguished two classes of ion channel (Fiorillo, 2008). Class 1 ion channels were proposed to contribute “current” sensory related information, which generally corresponds to the EPSG, whereas class 2 inputs contribute “prior information” that is “the prediction” in “prediction error”. The defining and categorical distinction was that class 1 ion channels (and synapses) have been selected through evolution or Hebbian learning to drive membrane voltage (or firing rate) away from the homeostatic level where the error is 0, whereas class 2 ion channels have been selected (through evolution or anti-Hebbian learning) to drive voltage towards its homeostatic level. The distinction is categorical (rather than merely denoting statistical tendencies) since implementation of one learning rule rather than its opposite would require that ion channel proteins are somehow “tagged” in a way that would determine whether the channel is to be regulated by either a Hebbian or anti-Hebbian rule (Fiorillo, 2008). The proposal that voltage-regulated ion channels can be regulated by associative learning rules remains unverified.

The present work provides further insight by specifying that the homeostatic level is spike threshold. GABA_A_-mediated IPSGs can be class 1 or 2, as illustrated by the two types of GABA synapse on thalamocortical neurons of LGN, both of which derive from local interneurons (Blitz and Regehr, 2005; Wang et al., 2007, 2011). Retinogeniculate excitation of an ON-type neuron provides evidence for high light intensity (or positive contrast), whereas excitation has the opposite meaning in an OFF-type neuron. In an ON-type neuron, inhibition from an OFF-type interneuron (with an almost identical receptive field) contributes evidence against high light intensity (and against the EPSG), as shown in Figure 3 (Wang et al., 2007). Sometimes there will be evidence both for and against light in the receptive field at the same time, but generally these will be anti-correlated (Mastronarde, 1989). Thus OFF-type inhibition will usually occur in the absence of EPSGs and drive hyperpolarization away from spike threshold (Figure 3). However, an ON-type neuron also receives synaptic inhibition from an ON-type interneuron (with a similar receptive field). This “same-sign” inhibition naturally occurs at nearly the same time as retinogeniculate excitation (typically delayed by 1 ms), and is caused in a feedforward manner by the same retinal ganglion neuron (Blitz and Regehr, 2005), and thus it is homeostatic and prevents excessive excitability. We did not incorporate opponent inhibition into our framework above, but we could do so by proposing that homeostatic mechanisms should predict the sum of new evidence for and against a neuron’s stimulus, and a neuron generates a spike when new evidence for its stimulus (an EPSG) exceeds both new evidence against it (opponent inhibition) as well as old evidence for it (homeostatic inhibition).

Sodium channels may also be class 1 or 2. Axonal action potentials are the “all-or-none” output of a neuron, and the sodium channels that cause them clearly drive voltage away from homeostasis and would be class 1. By contrast, it is generally believed that dendritic sodium channels do not consistently contribute to causing axonal action potentials (axonal spikes are not initiated in dendrites). If they usually amplify dendritic EPSPs towards threshold for an axonal spike under natural conditions, they would be homeostatic, whereas if they usually drive depolarization beyond threshold for an axonal spike, they would oppose homeostasis and be in class 1. It is interesting to note that class 1 and class 2 sodium channels could have identical structure at the level of individual channels, but their opposite relationship to homeostasis could be due solely to their sub-cellular localization and density.

## Sensory versus motor neurons

The principle of predictive homeostasis should apply to all neurons, and even all cells. However, we suspect that there is an important difference between sensory and motor neurons, and that above we have described sensory neurons. We do not try to justify the distinction here, but a rough and intuitive summary is that sensory neurons use a binary spike output to efficiently convey information about “shades of gray” in their sensory input (EPSG amplitude), whereas motor neurons use it to convert a shade of gray into a “black or white decision”. As partially summarized elsewhere (Fiorillo, 2013a), neurons in motor systems are proposed to express voltage-regulated ion channels (such as L- and T-type calcium channels) that drive voltage away from spike threshold (class 1 ion channels). This is known to be the case in some motor neurons (Marder and Bucher, 2007), but we propose it to be a general feature of motor neurons. In the case that these channels are excitatory, they would tend to be coactive with EPSGs, and together with EPSGs they would cause spikes. The goal of predictive homeostasis (class 2 ion channels) in motor neurons would be to maintain excitability by predicting the sum of EPSGs and these pattern-amplifying intrinsic conductances. Our definition of SP does not incorporate knowledge of the state of these intrinsic “drivers”, and we suspect that they would have the effect of driving SP towards the extremes (0 or 1) so that it is rarely near 0.5. Nonetheless, effective homeostatic mechanisms should still cause long-term average SP to be near 0.5. Furthermore, since EPSGs would not be the sole cause of spikes in motor neurons, spike count may outnumber EPSG count during some excitatory events.

## Implications of predictive homeostasis

### Metabolic cost of predictive homeostasis and spikes

The importance of predictive homeostasis is evident in the large amount of energy that it consumes. Sodium, potassium, and chloride conductances all tend to increase in synchrony, allowing these ions to flow down their electrochemical gradients and counteract one another electrically while causing only modest changes in membrane voltage (Pouille and Scanziani, 2001; Shu et al., 2003; Wehr and Zador, 2003; Blitz and Regehr, 2005; Hosoya et al., 2005; Wilent and Contreras, 2005; Berg et al., 2007; Okun and Lampl, 2008; Wang et al., 2011; Sengupta et al., 2013). The high cost that neurons pay demonstrates the biological value of the information that they gain from predictive homeostasis.

In proposing that SP of 0.5 is optimal, we are ignoring the metabolic cost of action potentials (Attwell and Laughlin, 2001). If their cost were high relative to the value of the information they convey, the optimal SP should be lower than 0.5 to conserve energy. Although we do not consider metabolic costs here, we do review experimental evidence below that SP is maintained moderately close to 0.5, consistent with the possibility that metabolic costs do not have a large impact on SP.

### The meaning of spike output to sub-cellular observers

A spike exerts its effect on sub-cellular observers (e.g., proteins) throughout the neuron. A spike can be approximated as a digital or binary signal, but it acts on virtually all neural observers as an analog signal, perhaps most notably in its effects on calcium concentration. If we were to analyze what these observers know about EPSG amplitude and what information is provided by the spike, we would follow the same framework given above. By predicting some local quantity like calcium concentration, they would also be predicting the more distal EPSG amplitude, which in turn corresponds to information about more distal quantities including a stimulus external to the nervous system.

Will the optimal “encoding strategy” of our neuron benefit these other observers, even though they are ignorant of the membrane excitability? The answer is “yes” because the more effective the predictive homeostasis, the less patterned the spike output, which would be “temporally decorrelated” relative to synaptic input (e.g., Wang et al., 2003; Deneve, 2008a). A spiking neuron with any other encoding strategy, or which makes inaccurate predictions, would have a less variable and more predictable output and thus convey less information from the perspective of most observers. This also applies to an observer who counts spikes over a longer period of time (a “rate code”). The fact that the firing rate of neurons is “analog” and not merely binary depends on the same sorts of homeostatic mechanisms, such as the potassium channels that are activated following a spike and create the “relative refractory period”. Without a relative refractory period, a neuron’s firing rate would be either 0 or a fixed number of spikes in response to any input (the number depending only on the absolute refractory period).

The benefit of maximizing the variance of input or output can be a source of confusion. It may seem that if the goal of an observer is to minimize its uncertainty (posterior entropy), it would be best to select inputs that have minimum variance and are thus more predictable. This issue has been called “the dark room problem”, since the uncertainty of neurons in the visual system would appear to be minimized if the animal simply avoids light (Friston et al., 2012). The reason that unpredictable inputs are better, other things being equal, is that the uncertainty that ultimately matters is not about EPSG amplitude, or the amount of light that enters the eye, or even about the identity of distant objects, but about future value. This explains the fact that animals explore uncertain environments, and the utility of Hebbian learning, which maximizes positive prediction errors (Fiorillo, 2008).

We favor the view that the flow of information follows the flow of force and energy and causation, implying that the absence of one event (a spike) is never the cause of another event, and therefore conveys no information. However, in the context of a force and energy that creates an expectation (e.g., a potassium current), the absence of an event (e.g., an EPSG) could allow the expectation itself to modify the observer’s knowledge (e.g., hyperpolarize the membrane). The occurrence of an EPSP would be known to some dendritic proteins, and the EPSP would cause the expectation of a spike. The absence of a spike in the context of an EPSP would indicate that the EPSP was smaller than expected. By contrast, an axon terminal (or its postsynaptic targets) does not experience an EPSP. Such an observer would have no means to distinguish whether the absence of a spike corresponds to no EPSP or an EPSP of less than expected amplitude, and thus absence of a spike would convey little or no information.

### Opponent (ON–OFF) representations

If the absence of a spike conveys little or no information to an axon terminal and its postsynaptic targets, and yet a system is to be sensitive to decrements as well as increments, there must be “opponent” neurons that represent the same stimulus dimension (like light intensity) but with opposite polarity. The ON and OFF neurons in the visual system are a well known example (Wang et al., 2011), and it is likely that the neural representation of reward value follows the same principle (Fiorillo et al., 2013; Fiorillo, 2013b). The value of opponent representations has long been recognized on the grounds that firing rates are highly “rectified”, but single spikes are “fully rectified”. Thus a spike in an ON neuron would be evidence of “high”, a spike in an OFF neuron evidence of “low”, and absence of a spike is not evidence at all.

### Prediction errors in learning

A spike functions as a “teaching signal”, and what the “student observer” learns will depend on whether or not that observer knows that an EPSG has occurred. Observers within a dendritic spine can and should have information about the occurrence and timing of three events: a local EPSG at that synapse, a global EPSP, and a spike. Given this information and the Hebbian objective at an excitatory synapse of maximizing positive prediction errors (spikes) (Fiorillo, 2008), if an excitatory synapse was active just prior to a spike, it contributed to a positive error and should be strengthened. If a local EPSG at that synapse failed to cause a spike, then it contributed to a negative error (or alternatively, we can say that it was not associated with a positive error), and thus it should be weakened. If the local synapse was inactive, then it bears no responsibility for a spike or its absence, and its strength should not be changed substantially. This proposed rule is spike-timing dependent and shares an essential feature of the rule proposed by Bienenstock, Cooper, and Munro (the “BCM rule”), in which synaptic activation paired with only modest postsynaptic activity causes depression of synaptic strength (Bienenstock et al., 1982). The BCM rule has useful computational properties and is supported by experimental evidence (Cooper and Bear, 2012). The BCM-like rule proposed here would allow a neuron to selectively strengthen those excitatory synapses that are least predictable and thus provide the neuron with the most new information (Fiorillo, 2008).

Whereas observers in the dendrite can gain information from a “spike failure” (EPSG + no spike), downstream observers in the axon terminal or its postsynaptic target neuron would not. For example, midbrain dopamine neurons signal a “reward prediction error” that is thought to teach reward value to downstream neurons (e.g., Schultz et al., 1997; Izhikevich, 2006; Fiorillo, 2008; Frémaux et al., 2010). Whereas past models used both increments and decrements in firing rate (positive and negative prediction errors relative to “spontaneous, baseline firing rate”), here we conclude that teaching should be performed by spikes alone, not their absence. Indeed, recent experiments have shown that decrements in firing of dopamine neurons are not useful for learning about “negative reward value” (Fiorillo et al., 2013; Fiorillo, 2013b). Rather, spikes from an opponent (“reward-OFF”) neuron would be essential for “bidirectional” learning.

### Comparison to other interpretations of spike variability

Whereas virtually all previous models have proposed that spike variability is indicative of greater uncertainty of neurons about their sensory inputs, we propose the opposite. Our optimal neuron fulfills Softky’s definition of an “efficient neuron”, having membrane excitability that is exquisitely fine-tuned to its synaptic input (Softky, 1995). Our ignorance of the neuron’s excitability (its internal knowledge) causes output that is maximally informative from the neuron’s perspective to appear random to us (Softky, 1995; Fiorillo, 2012).

The characterization of spike variability depends on probabilities, and we have previously criticized the field in general for failing to adequately distinguish conditional probabilities from event frequencies, and for failing to take the neuron’s point of view (Fiorillo, 2012). Spike variability is conventionally interpreted as “noise” within the literature on “efficient coding” (EC; Barlow, 1961; Rieke et al., 1997). Although we have come to the opposite conclusion, our interpretation is a straightforward application of information theory and it is generally in the spirit of EC.

We view our work, and the EC literature in general, as very distinct from a viewpoint that might be called “probabilistic computation” (PC), which is characterized by the assumption that neurons need to “represent and compute probabilities”. We agree with this viewpoint with respect to the general premise that the function of neurons is related to “Bayesian inference”, but we have a very different view of what this means (Fiorillo, 2012). First, opposite to our conclusion, a popular view within PC is that spike variability (in response to fixed inputs) increases with the subjective uncertainty of neurons (Pouget et al., 2000; Deneve et al., 2001; Hoyer and Hyvarinen, 2003; Jazayeri and Movshon, 2006; Ma et al., 2006; Beck et al., 2008; Berkes et al., 2011). Second, PC presumes that inference requires probabilities to be “calculated” by the brain, and thus neurons may perform methods like Expectation-Maximization (presuming that exact integration in calculus may be beyond the brain’s abilities) (Deneve, 2008a,b; Friston, 2010; Clark, 2013). Equations that describe reason, like Bayes’s Theorem, are viewed as prescriptive of what the brain should do. This is in sharp contrast to our view, and that of the EC literature and physics in general, in which probabilities are merely descriptive. We presume that, at least in principle, probabilities can be used by scientists to describe information that is physically integrated in both space and time (as in a neuron’s membrane voltage). This is precisely analogous to the way that calculus describes motion in physics. A moving object does not “perform calculus”, and the methods of calculus do not prescribe the motion of an object. The descriptive view is that it is sufficient to have the right information in the right place at the right time, without any additional need for “performing mathematics”. Biophysical models are purely descriptive, but PC and other theories that prescribe Bayesian principles for the brain have generally not addressed the fundamental issue of biophysical description (Fiorillo, 2012).

A particularly comparable example of PC is Deneve’s “Bayesian spiking neuron”, which signals prediction error (Deneve, 2008a,b). This model presumes that probabilities are a property of the neuron’s external stimulus, and that they are synaptically conveyed to a neuron. Thus a neuron’s membrane voltage and spike output represents probabilities (frequencies) of the stimulus, whereas our neuron represents the stimulus itself (its physical intensity). Beyond this fundamental difference, Deneve’s optimal neuron produces identical output in response to repeated presentations of identical input, whereas ours produces variable output (it would be impossible for any neuron to know if repeated inputs are identical, and we doubt that they ever can be identical in reality). The presumption of Deneve was that the null point, where the error is 0, should lie some distance below spike threshold (specified by the free and constant parameter “g_0_” in the model of Deneve). Thus if spike threshold is −50 mV, a perfect expectation might result in a membrane voltage of −55 mV, and would therefore never cause a spike in Deneve’s neuron. In our neuron, a perfect expectation would cause membrane voltage to be at spike threshold, thereby generating maximally variable output (SP = 0.5, similar to Deneve’s model if g_0_ is 0).

## Experimental evidence for predictive homeostasis

The theory predicts that homeostatic conductances should counterbalance EPSGs to maintain excitability, and that this should happen on all timescales, including the millisecond scale. The predictions of the theory can be made quantitatively precise given a particular EPSG pattern (Figure 2), as discussed above. In support of the theory, a remarkably precise balance of excitation and inhibition has been found in brain slices (Pouille and Scanziani, 2001; Shu et al., 2003; Blitz and Regehr, 2005) as well as *in vivo* in both sensory neurons (Wehr and Zador, 2003; Hosoya et al., 2005; Wilent and Contreras, 2005; Higley and Contreras, 2006; Okun and Lampl, 2008) and spinal motoneurons (Berg et al., 2007), even on a millisecond timescale (e.g., Wehr and Zador, 2003). Since this phenomenon is moderately well known we do not review it here, but instead focus on I-O relations.

### Temporal decorrelation and spike statistics

If a neuron makes accurate predictions and signals prediction error, its spike output should have less temporal pattern than its input, since the predicted patterns have been removed from the output. Multiple studies have demonstrated “temporal decorrelation” within sensory systems (e.g., Dan et al., 1996; Brenner et al., 2000), including at the level of single neurons (e.g., Wang et al., 2003, 2010b). Many additional studies have described spike statistics without such analyses of I-O relations. The present theory predicts that the more accurate a neuron’s predictions, the more random its spikes will appear (SP will be closer to 0.5). Thus neurons presented with dynamically varying inputs or complex patterns that are inherently more difficult to predict should have less random output than neurons exposed to more static conditions or simpler patterns. It is well known that many neurons have a relatively random, “Poisson-like” pattern of spike output under “resting conditions” (Tolhurst et al., 1983; Softky and Koch, 1993; Shadlen and Newsome, 1998; Churchland et al., 2010), which are relatively static by definition. By contrast, stimuli that are difficult to predict due to rapid temporal dynamics cause spike counts and intervals to be extremely regular across repeated trials (Mainen and Sejnowski, 1995; Bair and Koch, 1996; Berry et al., 1997; de Ruyter van Steveninck et al., 1997; DeWeese et al., 2003; Avissar et al., 2007; Butts et al., 2007). When the same stimuli are constant or more slowly varying, and thus more predictable, the same neurons show much greater variability in their spike statistics (Mainen and Sejnowski, 1995; de Ruyter van Steveninck et al., 1997; Butts et al., 2007).

### Thalamocortical neurons in LGN

Thalamocortical neurons in LGN offer several advantages for testing the theory. First, as part of the early visual system, they have been studied extensively both *in vivo* and *in vitro*, with respect to information theory as well as mechanisms. Second, they have a spiking input and output, like most neurons in the brain but distinct from some early sensory neurons such those in the retina. Third, they have one dominant presynaptic excitatory “driver” that generates large unitary retinogeniculate EPSPs (Usrey et al., 1998; Figure 3). This provides an important technical advantage, since the large size of unitary EPSPs allows them to be recorded simultaneously with spikes in a single neuron with a single extracellular electrode (Kaplan and Shapley, 1984). It also narrows the range of possible EPSG patterns, since there is little spatial summation. Furthermore, each unitary EPSG is of similar amplitude *in vivo* in LGN (Carandini et al., 2007; Wang et al., 2007), as well as in the case of other powerful synapses (Lorteije et al., 2009; Borst, 2010; Kuenzel et al., 2011). Thus we can approximate the input pattern by considering that it consists only of unitary EPSGs of equal amplitude, so the total synaptic drive is determined solely by temporal summation (Figure 2).

**Figure 4:**
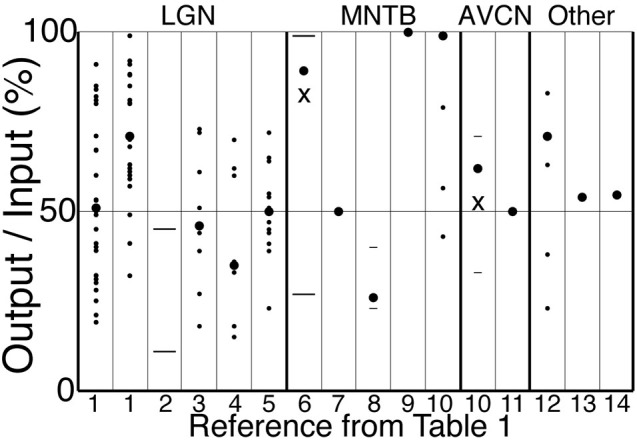
**Output to input ratios from the references in Table 1**. Symbols indicate mean across cells (large circles), individual cells (small circles), s.d. or quantiles (short lines), and range (long lines). For references 6 and 10, the “X” (mean) and other symbols correspond to a subset of cells in which O/I was less than 1, whereas large circles indicate our estimates of the total population mean. The strongest stimulus condition is shown for those references for which more than one condition is reported in Table 1. Reference 1 has data for cat (left) and monkey (right). A single cell with O/I of 150% is not shown for “12”.

Table 1 and Figure 4 show output and input rates from studies in LGN and elsewhere in which both were recorded simultaneously. Most studies reported only averages of SP over periods of seconds or minutes. We sometimes report averages of SP across populations of cells, but this is done only to provide a concise summary, or because greater detail was not reported in some studies.

**Table 1:** **Time averaged input and output rates and ratios from studies that measured both simultaneously over seconds or more in cells known to receive driving excitation from just one or a few presynaptic neurons**.

**Reference**	**Cells**	**Stimulus**	**N**	**Input (Hz)**	**Output (Hz)**	**O/I(%)**
1. Kaplan et al. (1987)	Cat LGN	Contrast 64-80%	37	NA	NA	51±20, 19-91
	Monkey LGN	Contrast 64-80%	26	NA	NA	71±17, 32-99
2. Movshon et al. (2005)	Monkey LGN	Contrast	7	NA	NA	11-45
3. Sincich et al. (2007)	Monkey LGN	Contrast	15	29±9 (n = 13)	13*	46±16
4. Casti et al. (2008)	Cat LGN	Contrast	14	NA	NA	35±19, 7-70
5. Weyand (2007)	Cat LGN	Contrast	12	50±16	24±8	50±13
6. Lorteije et al. (2009)	Mouse MNTB	Spontaneous	11	71±11, 0.4-174	62*	87, 32-99
		Monaural tone, 80 dB	15	352±34	289*	82, 27-99
7. Hermann et al. (2007)	Gerbil MNTB	300 Hz Elec. Stim.	18	300	150*	50*
8. Kopp-Scheinpflug et al. (2003)	Gerbil MNTB	Spontaneous One tone	54	49 [17-79] 1-308 355 [212-486] 48-868	21 [6-45] 0-189 194 [126-230] 18-334	48 [36-71] 5-94 55 [38-74 23-95
		Two tone		NA	NA	26 [23-40]
9. McLaughlin et al. (2008)	Cat MNTB	Binaural tone	49	100-500*	100-500*	100
10. Englitz et al. (2009)	Gerbil MNTB	Spontaneous	3	NA	NA	79, 39, 43
	Gerbil AVCN	Spontaneous	27	NA	NA	76±17
		1 & 2 tone monaural	34	NA	NA	52±19
11. Kuenzel et al. (2011)	Gerbil AVCN	Spontaneous	39	79*	56±23	71±26
		Monaural Tone, 51±11 dB	39	NA	NA	50*
12. Chadderton et al. (2004)	Rat cerebellar granule cells	Tactile, Ipsilateral	5	3.2* EPSCs	2.2* spikes	71, 23-150*
13. Bratton et al. (2010)	Rat lumbar SG	Spontaneous	38	5.4	2.9±0.3	54*
14. McAllen et al. (2011)	Rat cardiac SG	Spontaneous	6	4.1±0.7	0.2-4.3	55*

Portions of these data are illustrated graphically in Figure 4. The output to input ratio (O/I) should match average SP in principle, but could differ due to a technical failure to detect an input, especially in cases of high frequency bursts of input. Only “12” reported a single cell in which output exceeded input, but “12” was also the only study in which input and output counts were not measured simultaneously. None of these studies reported a significant incidence of either spikes in the absence of input, or more than one output spike resulting from a single input spike. Data are number of neurons (N), population mean±sd (median and quantiles for “8”) and range, except for 3 single cell’s in MNTB for “10.” Asterisks (*) indicate our estimates based either on our calculations or visual inspection of figures. NA: not available. “Spontaneous” indicates that sensory conditions were not specified and may not have been controlled. Studies were in anesthetized animals except “5” in awake cats, and “7” and “14” in vitro during efforts to mimic natural conditions. Data was obtained with extracellular recordings, except intracellular recordings in 6–7 and 12–14. In “6” and “10,” O/I are only presented for those neurons that displayed “failures” (O/I < 1.0), whereas O/I was reported as 1.0 in the remainder of 24 total neurons in “6,” and in 63, 21, and 9 other cells in “10” in the cases of spontaneous activity in MNTB and AVCN, and sound evoked activity in AVCN, respectively.

**Figure 5:**
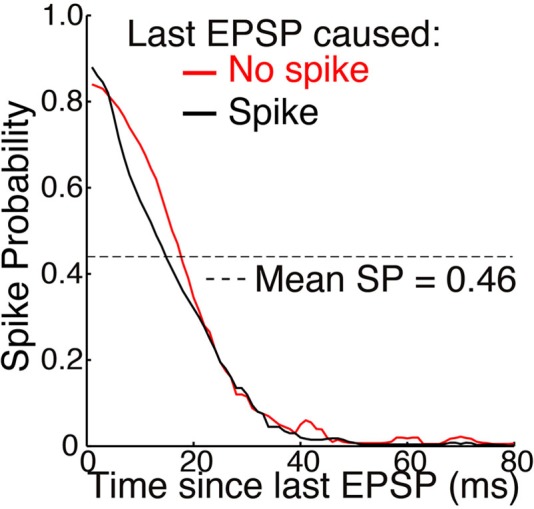
**Mean SP across 15 neurons as a function of time elapsed since the last EPSP (adapted from Sincich et al., 2007, with permission of the authors)**. Simultaneous extracellular recordings were made of both retinogeniculate EPSPs and the spikes they caused in individual neurons in anesthetized macaques. A spot of light or dark filled the receptive field center and varied with naturalistic patterns of contrast. Standard deviations of about ±0.25 were removed from the original figure. A spike caused no significant effect on SP across the population of neurons. Mean SP across the 15 neurons, and across time, was 0.46 ± 0.16 (see Table 1 and Figure 4).

Retinal spikes are the cause of all spikes in LGN (Kaplan and Shapley, 1984), and it is well established that average SP is near 0.5 at the retinogeniculate synapse *in vivo* (Kaplan et al., 1987; Movshon et al., 2005; Carandini et al., 2007; Casti et al., 2008), including when naturalistic stimuli are presented (Sincich et al., 2007) and in the awake state (Weyand, 2007). Casual inspection of whole-cell voltage recordings suggests that average SP has intermediate values even over periods of about a second (Figure 3; Wang et al., 2007). At least 95% of all spikes in LGN neurons were caused by retinogeniculate EPSGs (Sincich et al., 2007; Weyand, 2007). When an EPSG followed a long period without one, it rarely caused a spike (SP < 5%). However, following a first ESPG, SP at the time of a second rose to a population average of about 85% at 2 ms, and then declined back to its low “resting level” over 40 ms (Figure 5; Sincich et al., 2007). Weyand (2007) found similar results. There is strong evidence that SP over this time scale in LGN is influenced by fast homeostatic mechanisms (Blitz and Regehr, 2005), but in principle these data can be explained by temporal summation alone (Carandini et al., 2007; Casti et al., 2008) if cells have a nearly optimal leak conductance (Figure 2).

Sincich et al. (2007) also found that SP was not altered following a spike (Figure 5). This supports our theory, according to which opening of potassium and other channels following an action potential helps to maintain homeostatic excitability rather than causing hyperpolarization (see Mechanisms and Timescales).

Given sufficient knowledge of natural patterns, the theory should allows us to predict membrane excitability and the properties of specific ion channels. In work presented in preliminary form and currently under review for publication, we provide evidence in brain slices of LGN that T-type Ca^2+^ channels promote predictive homeostasis by restoring SP towards 0.5 during naturally occurring periods of hyperpolarization like that illustrated in Figure 3 (Hong et al., 2013). Without predictive amplification of EPSPs by T-type channels, SP would be near 0 for extended periods and spike output would be temporarily “blind” to visual stimuli. In support of theory, we found that the amplification of retinogeniculate EPSPs by T-type channels typically results in just 0 or 1 spike. Previous reports of T-type driven bursts of multiple spikes were most likely due to the unnaturally high membrane excitability that is typical of experimental conditions in brain slices.

### Evidence from other types of neurons

In the medial nucleus of the trapezoid body (MNTB) in the early auditory system, the calyx of Held is an exceptionally powerful synapse in which a single presynaptic terminal wraps almost entirely around the postsynaptic soma. It was long believed to be a reliable “fail-safe relay” with SP very near 1. However, the existence of powerful synaptic inhibition suggests that SP should sometimes be lower (Awatramani et al., 2004). SP has been found to be intermediate (Kopp-Scheinpflug et al., 2003) or almost 1.0 (McLaughlin et al., 2008; Englitz et al., 2009) based on extracellular recordings, but the more definitive technique of whole-cell patch recording found SP across cells to be near 0.9 *in vivo* with a monaural single tone (Lorteije et al., 2009). When attempts were made to mimic *in vivo* conditions within brain slices, SP varied across a range of about 0.2–1.0 centered near 0.5 (Hermann et al., 2007). None of the *in vivo* studies attempted to examine natural patterns of sound, and most used monaural or binaural pure tones (Table 1). Pure tones and monaural stimulation are rare events in the natural world. Sound intensities are usually similar across tones (“broadband”) and in both ears, and this likely provides the basis for homeostatic synaptic “surround” inhibition that suppresses SP, as found in many other neurons. Indeed, SP in response to the neuron’s preferred tone was suppressed (below 0.4) by simultaneous presentation of a second tone (Table 1; Kopp-Scheinpflug et al., 2003).

The endbulb of Held in the anteroventral cochlear nucleus (AVCN) is another powerful axosomatic synapse, and SP has been found to be near 0.5 during acoustic stimulation (Englitz et al., 2009; Kuenzel et al., 2011). Further support for the theory comes from the observation that as EPSP amplitude declined as a function of inter-EPSG interval over a period of milliseconds (apparently due to presynaptic decay of paired-pulse facilitation), excitability (and SP) increased in a corresponding manner so that spike generation adapted its sensitivity to the smaller range of EPSP amplitudes (Kuenzel et al., 2011).

That even the most powerful axosomatic synapses appear to have average SP nearer to 0.5 than 1.0 under natural conditions supports the theory, and makes it doubtful whether average SP is near 1.0 at any synapse. The climbing fiber to Purkinje cell synapse in the cerebellum is reliable (SP = 1) under standard conditions in brain slices, but the strength of other powerful synapses has been found to be much weaker under natural conditions *in vivo*, due to both pre- and postsynaptic factors (see Borst, 2010, for a review of presynaptic factors). We did not find any reports of stable, simultaneous recordings of pre- and postsynaptic activity *in vivo* at the climbing fiber to Purkinje cell synapse.

A typical cerebellar granule cell has four excitatory synapses of approximately equal strength, whereas sympathetic ganglion neurons are autonomic motoneurons that receive synaptic excitation from about 2–5 cholinergic preganglionic neurons, one of which is typically the main driver. Intracellular recordings have found SP to be near 0.5 in each of these cell types (Table 1; Figure 4; Chadderton et al., 2004; Bratton et al., 2010; McAllen et al., 2011).

In cells with larger numbers of drivers, such as cortical neurons, unitary EPSPs in the soma are typically not more than 1 mV, and significant spatial summation across synapses is required to evoke a spike. According to the theory, small unitary EPSPs should not be common under natural conditions, since that would cause SP to be very low. Indeed, there can be a high degree of synchronous excitation causing large EPSPs in at least some cortical neurons (DeWeese and Zador, 2006; Poulet and Petersen, 2008; Okun and Lampl, 2008; Gentet et al., 2010; Yu and Ferster, 2010), as suggested by simulations and theory (Softky and Koch, 1993; Wang et al., 2010a). In auditory cortex, extracellular recordings of spikes (DeWeese et al., 2003) and intracellular recordings of EPSPs with spikes blocked (DeWeese and Zador, 2006), were each performed under otherwise similar conditions. Taken together, these latter two studies imply that large EPSPs cause spikes with intermediate SP.

We have summarized above all of the highly relevant data that we found from simultaneous recordings of synaptic drive and spike output, and it supports our hypothesis that average SP is near 0.5 under natural conditions. However, of the modest number of *in vivo* studies that are most relevant to our hypothesis, few reported direct and detailed analyses of SP, and few attempted to study natural patterns of synaptic excitation.

## Vesicle release probability at presynaptic terminals

When a spike arrives at a single release site in a presynaptic axon terminal, it typically causes release of just 0 or 1 vesicle. Release is commonly said to be “stochastic”, but it is a highly regulated process (Branco and Staras, 2009), and the appearance of “randomness” reflects our ignorance of the internal machinery that determines whether or not a vesicle is released (see Fiorillo, 2012). It seems almost inconceivable that biology could not have achieved reliable transmission if it were desirable. We propose that vesicle release is analogous to spike generation, and that it only occurs when spike input exceeds its expected value. Since a neuron’s spike conveys information that is qualitatively identical to its EPSG input (it shares the same receptive field and thus concerns the same part of the world), it may seem redundant and pointless to repeat in the axon terminal the same sort of process that occurs in the dendrites and soma. However, an axon terminal is generally far from the soma, and we presume that there is information delivered to the terminal from local neurons (for example, via GABA). In principle this information could be conveyed to the soma, but that would consume time and energy, and it is thus more efficient to use it locally to predict incoming spikes, as previously suggested (Fiorillo, 2008).

Spikes entering a terminal from the axon would presumably be the sole driver and proximal cause of almost all vesicle release. They act via a transient increase in calcium conductance (analogous to an EPSG) and cytosolic concentration (analogous to an EPSP). The free calcium concentration varies from spike to spike and depends on a variety of factors, including the state of potassium channels (that influence action potential duration), calcium channels, and calcium buffers (Meinrenken et al., 2003). A vesicle that is “docked” and “ready” will be released in the event that calcium concentration exceeds a threshold (created by the requirement for binding of multiple Ca^2+^ ions to proteins that drive vesicle exocytosis). “Vesicle releasability” (analogous to membrane excitability) would be defined as the energy barrier separating the state of the release site from release threshold. In analogy to Hypothesis 1, it would correspond to the expectation at the onset of a spike of the peak calcium conductance (which would typically be reached a fraction of a millisecond later). Vesicle release would therefore only occur when the expectation is exceeded. Since we cannot directly observe releasability, we define release probability (RP, more commonly abbreviated “P_r_”) as the probability that an action potential will cause release of at least one vesicle at a single release site. As in the case of SP, RP reflects our ignorance of the internal state of the synaptic terminal.

If we could measure RP in real time at a single release site, then we would expect it to have the general features of SP, with numerous processes of excitation and inhibition (“facilitation and depression”) acting on numerous timescales. The goal of predictive homeostasis would be to make accurate predictions of calcium conductance, with the result that vesicle release is maximally sensitive to small variations in the amplitude of calcium events (conductance and concentration). A perfectly accurate expectation would result in RP of 0.5. Like SP, we would expect RP to vary dynamically from nearly 0 to 1 with a homeostatic average of 0.5.

Average RP (across release sites and over time) has been found to fall within an intermediate range (∼0.1–0.9) with an “average of averages” near 0.5 across a diversity of synapses (Branco and Staras, 2009). However, measures of RP are typically performed in brain slices in the presence of unnaturally high extracellular calcium and low firing rates (Hermann et al., 2007; Borst, 2010). A more general obstacle is that synaptic terminals are relatively poorly understood, mostly due to their small size, and this limits our ability to “take the release site’s point of view”. If we had a model of vesicle release comparable to a Hodgkin-Huxley model of membrane voltage, and the ability to monitor local calcium concentration at a single release site on a millisecond timescale, then we would not need to rely so heavily on the crude and indirect measure of RP. Typical measures of RP are less direct than what we have presented above for SP, since they are usually averages over many release sites, sometimes involving multiple presynaptic neurons.

We would like to know RP as a function of ISI, analogous to SP in Figure 5. However, studies often track the dynamics of RP (through its influence on EPSG amplitude) without quantifying its actual value. Because calcium concentration depends strongly on ISIs, and predictions should be accurate in the case of commonly occurring ISIs, we would expect that RP should be particularly sensitive to variations near these typical intervals. Indeed, in neurons of the fish electrosensory system that have average firing rates near 200 Hz, RP is highly sensitive to ISI variations near the average of 5 ms (Khanbabaie et al., 2010).

A unitary EPSG usually involves multiple release sites, and the powerful synapses discussed above have hundreds. Thus a presynaptic spike will almost always cause an EPSG, and its amplitude will be determined by the average RP across multiple release sites. Because homeostatic mechanisms at each release site are all working independently to “pull” RP towards 0.5, and because each release site experiences the same pattern of spikes, average RP across a presynaptic neuron’s synapses (and the corresponding postsynaptic EPSG amplitude) should not show large variations over time (depression and facilitation should be modest). This has indeed been found to be the case *in vivo* (Borst, 2010). However, maintaining average RP near 0.5 across release sites would maximize the variance (over time and release sites) in RP and unitary EPSG amplitude relative to any other average RP. The resulting variations in EPSG amplitude should convey information, and effective homeostasis of excitability in the postsynaptic neuron would maximize the sensitivity of spike output to these variations (and thus to presynaptic RP). In support of this theory, it has been found that although variations in presynaptic RP and postsynaptic EPSG amplitude are modest, spike output adapts to maintain high sensitivity to them (Kuenzel et al., 2011).

## Conclusion

We believe that taking the neuron’s point of view is essential if we are to understand the relation between biophysical mechanisms and their information content. Perhaps the most striking insight provided by our approach is its simple explanation of why spikes appear so “random”. What appears noisy and even pathological from the standard perspective of a physiologist can be readily understood as optimal from the perspective of a neuron. Taking the neuron’s point of view is a radical departure from past efforts to understand the neural “code”, and it could provide the foundation for a general theory of brain function that is able in principle to explain and predict the diversity of neural mechanisms (Fiorillo, 2008, 2012; Hong et al., 2013).

## Author contributions

Christopher D. Fiorillo conceived the ideas and wrote the manuscript. Jaekyung K. Kim performed the simulations shown in Figures 1 and 2, and Jaekyung K. Kim and Christopher D. Fiorillo designed Figures 1 and 2. Su Z. Hong and Christopher D. Fiorillo reviewed the experimental literature on SP and designed Table 1 and Figure 4.

## Conflict of interest statement

We have cited a patent that discusses the distinction between sensory and motor neurons within its specification (Fiorillo, 2013a). The patented material itself concerns application of Hebbian learning rules to voltage-regulated ion channels, and the patent was not cited with respect to issues of learning.
